# Case Report: A digital workflow in the treatment of bruxism in a young patient

**DOI:** 10.12688/f1000research.72961.1

**Published:** 2021-09-07

**Authors:** Dobromira Shopova, Krasimir Mladenov

**Affiliations:** 1 Department of Prosthetic Dentistry, Faculty of Dental Medicine, Medical University, Plovdiv, 4000, Bulgaria; 2 Center for Dental Medicine, Kyustendil, Bulgaria

**Keywords:** splint, TMJ disorders, bruxism, Exocad, CeramillMind, digital technologies

## Abstract

Bruxism is increasingly common in today's stressful world and affects mainly young patients. It is a combined disease that involves dentition and its supporting structures, muscles, ligaments and the temporomandibular joint (TMJ). Here we present a complete combined analog and digital clinical protocol in a patient with parafunction.

A young patient sought help due to impaired aesthetics, as a result of abraded tooth surfaces and severe symptoms of TMJ. We implemented a therapeutic protocol of six stages: deprogramming of the muscles and determination of treatment position and digital optimization; realization of the morphological plan for the upper dentition; non-invasive repositioning of the lower jaw by splint therapy; splint placement and follow-up; morphological planning of the lower dentition and replacement of the splint with fixed prosthesis with follow-up; and completion of the case with ceramic restorations.

The digitally modeled temporary constructions for the upper jaw were made of PMMA and placed in the patient's mouth together with the splint on the lower jaw, made of Ceramill Splintec. After an adaptation period, all restorations were replaced by permanent zirconia.

We achieved restoration of the defects of the dental arches and hard dental tissues and recovery to normal height of the lower third of the face (vertical dimension occlusion), fixed a stable and balanced position of the lower jaw, and repaired the normal physiological position of the TMJ for the patient. Аfter a multi-stage treatment we received a result satisfying the patient, the dentist and the dental technician. Aesthetics and function were restored, and clinical symptoms were removed from the TMJ.

## Introduction

In today’s stressful world, there are more and more diseases whose triggering or supporting component is stress. Bruxism is a parafunction with the basic etiological factor of stress. Other causes of bruxism can be occlusal disbalance or trauma. ^1^ Muscle hyperactivity is a proven concomitant factor in bruxism. One of the goals of bruxism treatment is to overcome the influence of the muscles by fixing the lower jaw in a stable position, i.e. a musculoskeletally stable position. ^1, 2^ Joint problems in the temporomandibular joint (TMJ) can be manifested by popping, clicking, limited opening, difficulty in opening and/or closing, and hearing problems. This symptomatology has a multifactorial origin - congenital malformations and syndromes, rheumatoid arthritis, fractures of the condyle, disc displacement, fibrosis, bony ankylosis, etc. Very often, the main symptom that makes the patient seek help is pain. ^3, 4^

As a consequence of bruxism, abraded tooth surfaces are observed, which correspond in shape to the antagonists. With directed movements, very accurate reproduction of pathological unconscious movements is possible. Most patients can reproduce the pathological movements on request and the abstracted surfaces are clearly visible. These surfaces slide on a plane, not at a point as is normal, and fit perfectly in shape. Depending on the degree of occlusal tooth abrasion, there are different phases: initial (Attritio dentis), advanced (Attritio dentis cum abrasione) and final (Abrasio dentis). ^5, 6^ Occlusal tooth abrasion may not be the result of parafunction. Predisposing factors can be divided into external causes (excessive use of carbonated beverages, citrus fruits, work in adverse conditions, etc.) and internal (gastro-oesophageal reflux, bulumia, congenital weakness of the enamel, etc.). ^7, 8^

According to Okeson, splint treatment options include two main types: stabilization splint and repositioning splint. ^1^ Four additional types of splints with specific indications are described by another authors: pivot splint, soft splint, anterior bite splint, posterior bite splint. ^9, 10^ Bumann an Lotzmann also use the terms relation splint, whose purpose is to normalize the tonus of the muscles of mastication by equalizing posterior tooth contacts; decompression splint, which should be made to fit the centric jaw relation existing at the moment ("momentary centric"); and verticalization splint, which increases the vertical dimension of occlusion (VDO). ^3^ A recent study on the effectiveness of anterior repositioning splints has shown an impact on dental anomalies. The splints are constructed by sliding the lower jaw forward to a Class I position in the area of the molars and at a distance of 5 mm between the premolars. During this movement, the joint disc stands in the correct position, which is maintained even after the end of the treatment. ^11^ Balancing contacts are desirable from a therapeutic standpoint because they reduce the load on the joint surfaces. ^3^ Anterior positioning appliance should accurately fit the maxillary teeth with total stability and retention when in contact with the mandibular teeth and when checked by digital palpation. ^1^

With the development of dental medicine, dental software for digital information processing has appeared. Splint design differs between patients, but for most the main features allow canine guidance, anterior guidance, occlusal relief, flat plane, and raise ramp. ^12, 13^ A digital design allows very precise outlining of the boundaries of the splint, a change of the intermaxillary relationships, change of VDO, and balanced occlusion. The option to use the virtual articulator allows visualization of the movements of the lower jaw, as well as the removal of pre-contacts, if necessary. ^14, 15^ The resulting version of the splint can be made by 3D printing or CAD/CAM milling. The technological characteristics of the objects made by cutting (CAD/CAM technology) from the monolithic block are higher and preferable than 3D printing. ^16^ CAD/CAM technology allows the production of extremely accurate and aesthetic structures. The digital design leads to a predictable result, approved by the dentist and the patient before its actual production. Zirconia-based ceramics combine very good mechanical strength and technological characteristics, which are complemented by aesthetics. ^17, 18^

Here, we demonstrate a combined analog and digital clinical protocol for the treatment of a patient with parafunction. The case is very complicated and complex. It requires good collaboration between the dentist and the dental technician, and great cooperation and patience on the part of the patient. To achieve this goal, digital methods of treatment have been used, which are not part of everyday practice. Some of the applied methods have not been described so far.

## Case report

### Initial presentation

A 35-year-old female patient, Bulgarian restaurant owner, sought help due to impaired aesthetics, as a result of abraded tooth surfaces and severe symptoms of the right TMJ.

**Figure 1.**
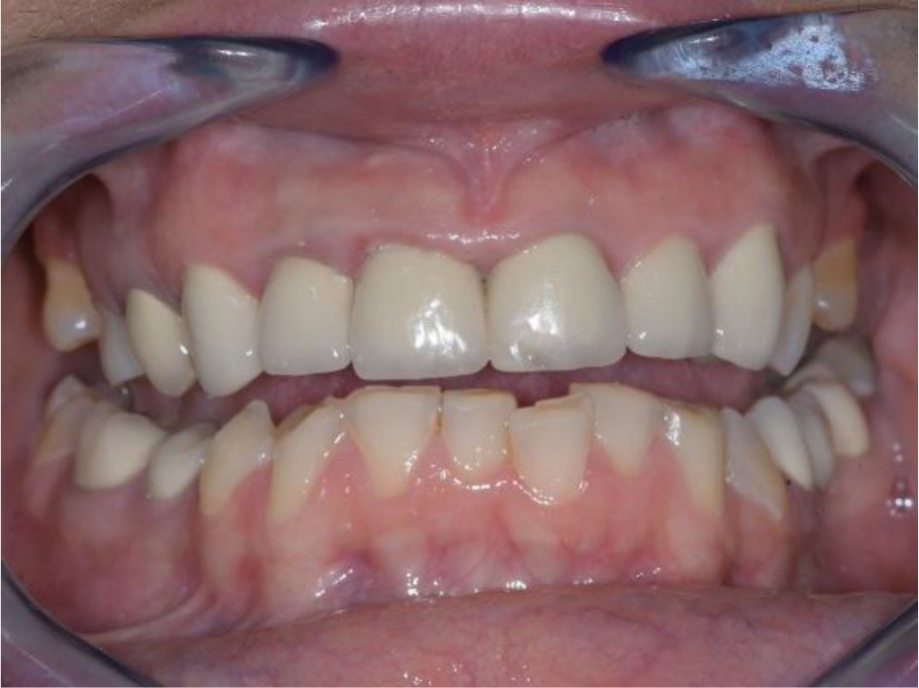
Initial clinical situation.

**Figure 2.**
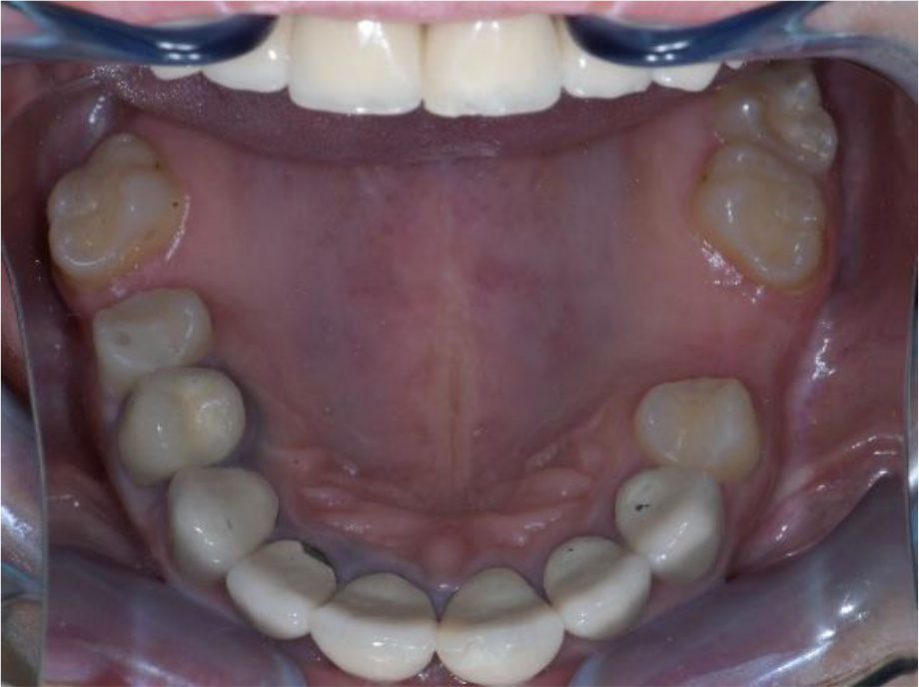
Upper jaw at initial presentation.

Intraoral examination of the upper jaw revealed a metal-ceramic construction on 13, 12, 11, 21, 22, 23, 24, missing 18, 16, 25, 26. The metal-ceramic construction was made 13 years ago, and was in good condition except for small fractures of the ceramic on the palatal surface and palatal staining of the gingiva by the metal base. The distal available teeth were medialized ( Figures 1 and 2).

**Figure 3.**
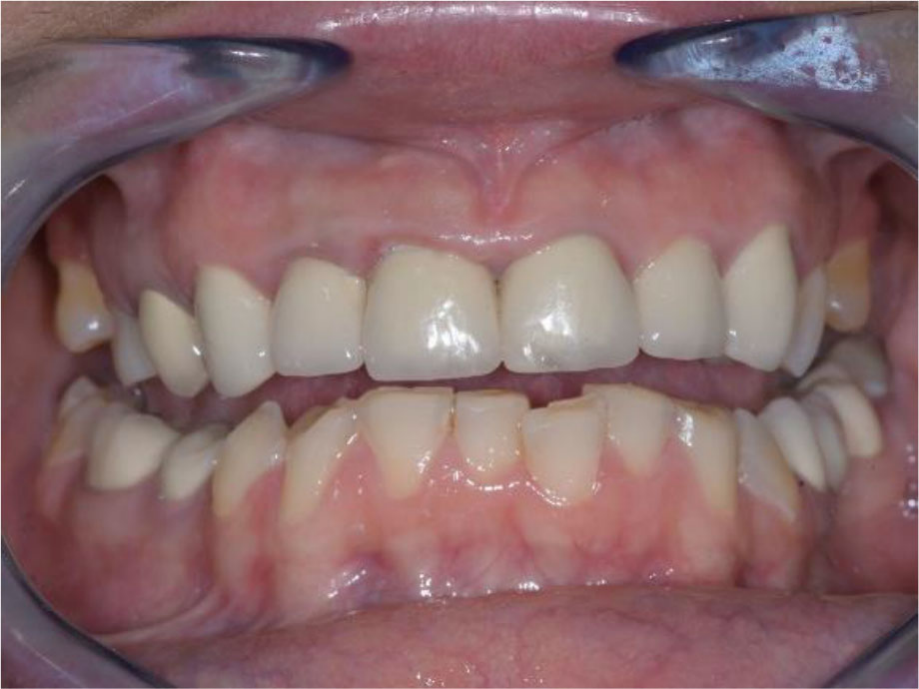
Abraded lower teeth and occlusal plane at initial presentation.

**Figure 4.**
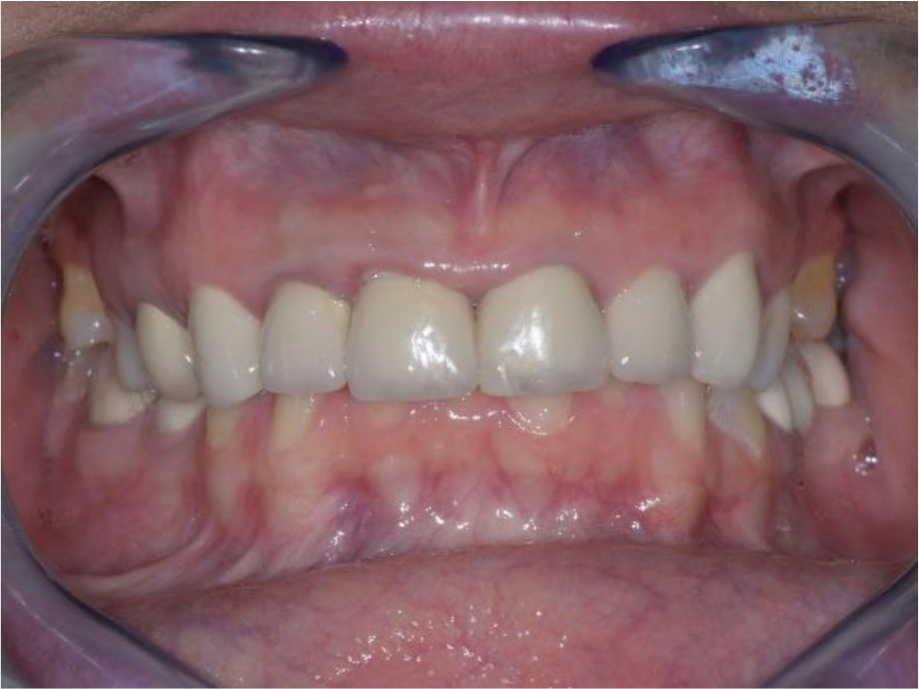
Central occlusion shown on initial presentation.

On the lower jaw, an advanced phase of abrasion (Attritio dentis cum abrasione) of the frontal teeth was established, reaching the dentin area. Defects of the dentitions were solved with ceramic restorations of 35, 36, 37 on the left and 45, 46 on the right. The occlusal plane was incorrect – the lower frontal teeth were higher. There was a deep overlap of the lower frontal teeth in central occlusion ( Figures 3 and 4).

During palpation of the TMJ, the patient responded with mild pain on the right side.

**Figure 5.**
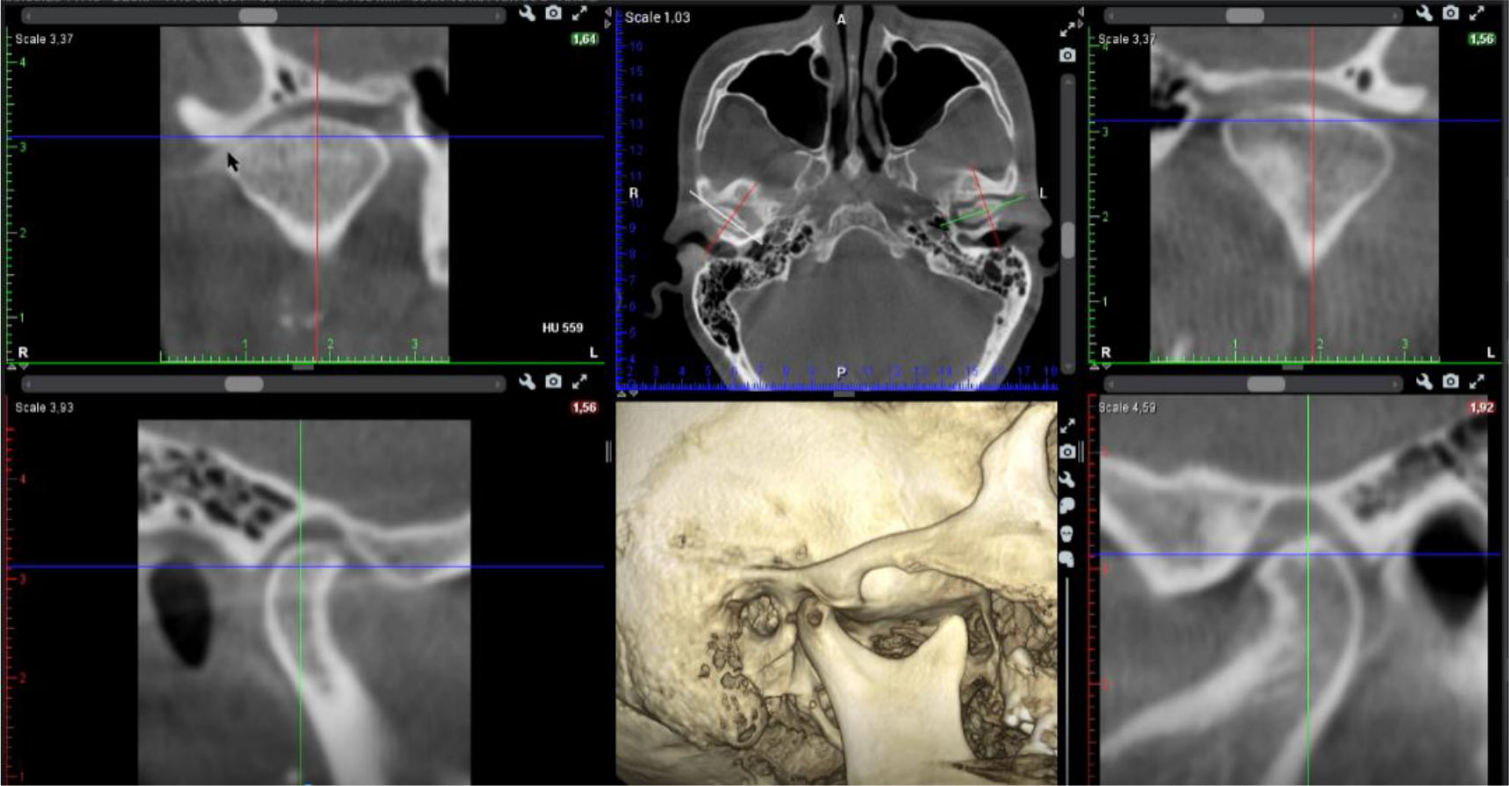
CBCT visualization – right and left TMJ.

**Figure 6.**
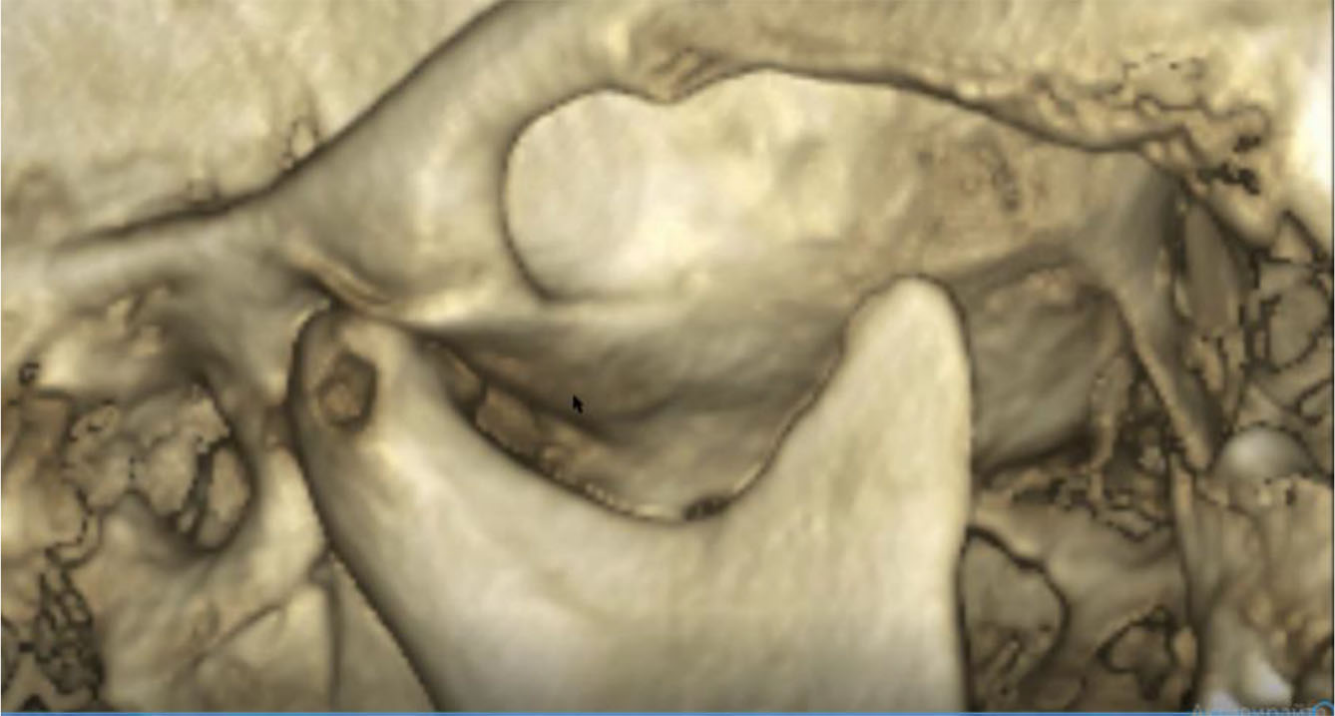
Right TMJ condition with osteophyte.

A cone beam CT (CBCT) scan showed an intra-articular problem in the right TMJ. The processus condylaris mandibulae had a preserved convex shape, but its lateral side was in contact with the eminentia articularis. The joint gap between these structures was asymmetric - wider medially and narrower laterally. In the medio-distal direction, the articular condyle was positioned more distally than the normal physiological position. The presence of osteophyte laterally was also established. The left TMJ did not show deviations from the norm ( Figures 5 and 6).

### Proposed therapeutic protocol

The therapeutic protocol included treatment in six stages:

• Stage 1 - deprogramming the muscles, determining the treatment position and digital optimization of the working position
• Stage 2 - realization of the morphological plan for the upper dentition
• Stage 3 - non-invasive repositioning of the lower jaw by splint therapy
• Stage 4 - splint placement and follow-up
• Stage 5 - morphological planning of the lower dentition and replacement of the splint with follow-up
• Stage 6 - completion of the case with ceramic restorations.

*Stage 1: Deprogramming the muscles, determining the treatment position and digital optimization of the working position*

**Figure 7.**
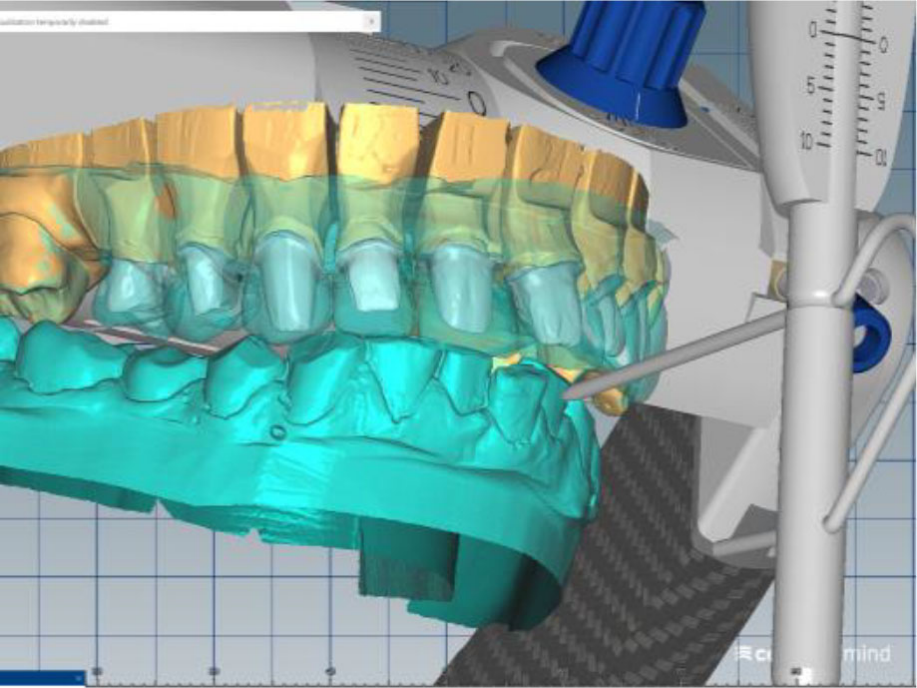
Digital design – increased VDO.

**Figure 8.**
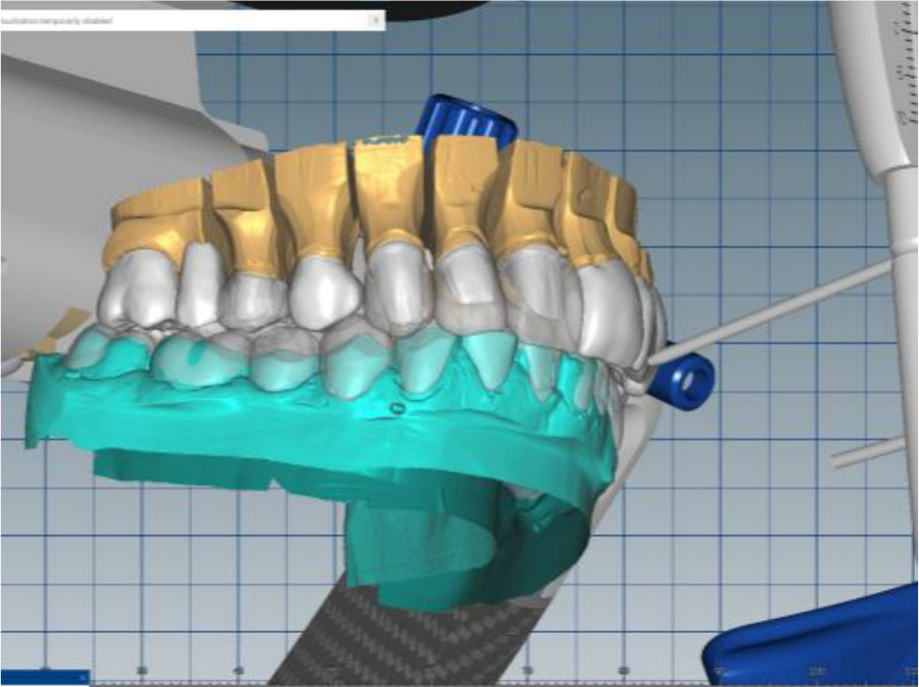
Digital design – crowns.

Silicone impressions and intraoral scanning of the patient’s initial clinical situation were performed with a Medit i500 scanner. AmannGirrbach’s facebow was used to capture the position of the dentition relative to facial landmarks - midline, interpupilary line, Camper plane and more. This information is transmitted to the laboratory and the resulting structures are symmetrical and horizontal. Digital planning and design of the splint was done through Exocad and CeramillMind, in which the models were also included in a virtual articulator. The software does not take into account the influence of the muscles closing the lower jaw (deprogramming the muscles). Thus, the upper and lower jaws can move in space - opening, protrusion, retrusion, laterotrusion. After deprogramming the muscles, a treatment position was registered, independent of the interdental contacts, with a new clinically defined VDO, the so-called static register of the real system. When measuring the volume, a levitation of 6 mm was found. The stage was completed with digital optimization and determination of the working position of the lower jaw ( Figures 7 and 8).

*Stage 2: Realization of the morphological plan for the upper dentition*

Shoulder preparation of the upper dentition was performed, and two-phase two-stage silicone impressions were taken ( *ZetaSoft + Oranwash, Zhermack*). The intermaxillary relationships were recorded by hard material ( *Protemp 4*).

**Figure 9.**
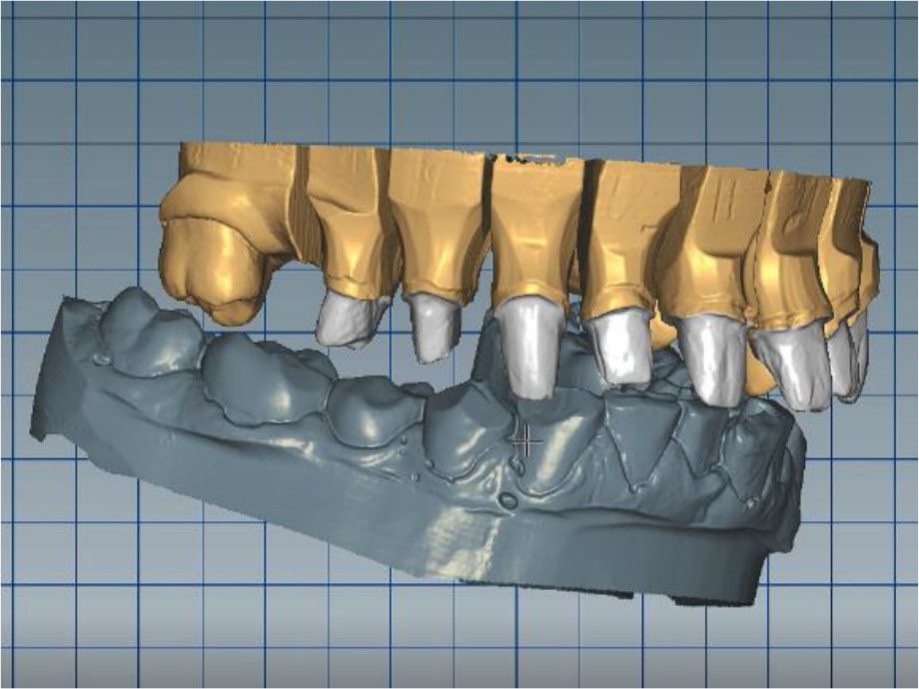
Clinical situation after upper preparation with increased VDO.

**Figure 10.**
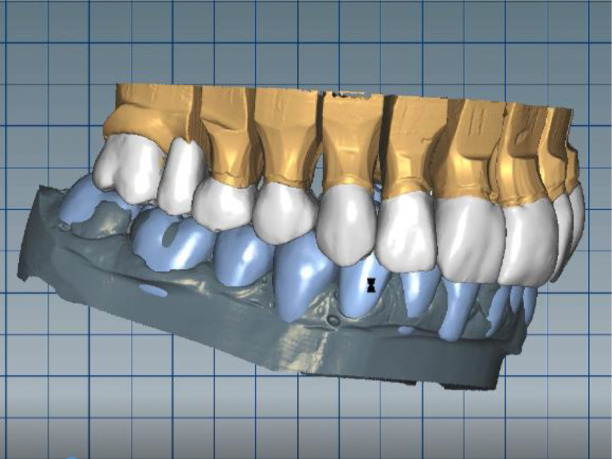
Digital design on both jaws.

The cast gypsum models were scanned with a laboratory scanner and the obtained digital models were subjected to additional processing. Briefly, transfer of the morphological planning for the upper dentition (crown strategy) and for the lower dentition (digital wax up strategy, pontics) was performed ( Figures 9 and 10).

**Figure 11.**
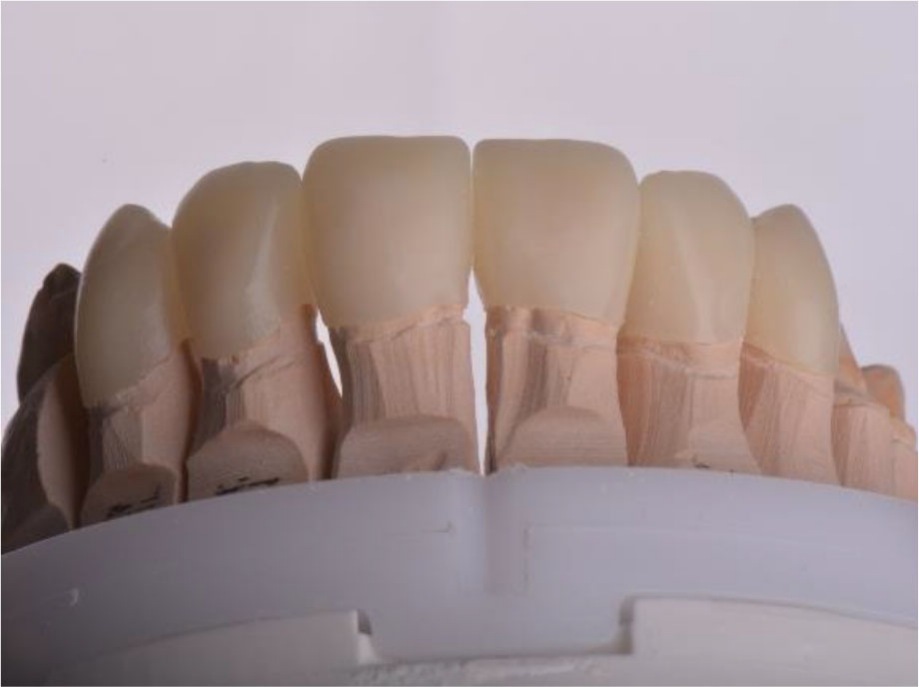
PMMA crowns on a model.

**Figure 12.**
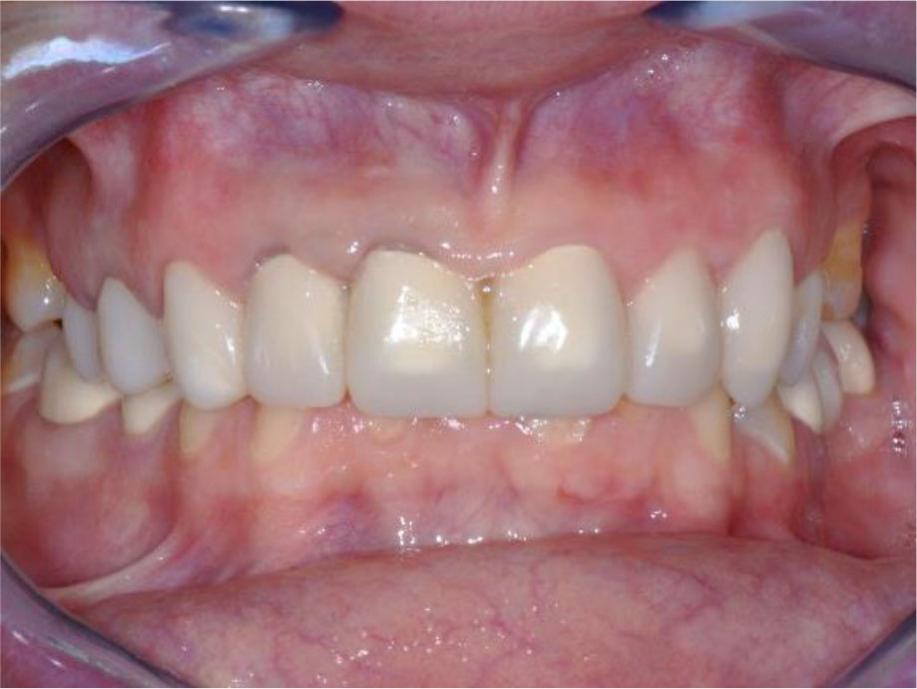
PMMA crowns cemented in the mount.

For the adaptation period, it was preferred that the temporary construction of the upper jaw be made of PMMA ( *Ceramill TempML*), which was cemented with temporary cement ( *Prevision (Kulzer)*). This construction was intended to remain in the patient’s mouth throughout the period of adaptation and, if necessary, to be optimized ( Figures 11 and 12).

*Stage 3: Non-invasive repositioning of the lower jaw by splint therapy*

The position of the lower jaw was optimized. A new working position was measured – normal central relation of the patient was increased with 6 mm (VDO raised with 6 mm). A tendency for compression in the right joint was established (the articulator was used as a variator). The position of the mandibular model was corrected by distraction of the right joint by 1.5 mm and medialization of the lower jaw by 1.5 mm. After the new adjustments, a new inclusion in the articulator and repositioning of the digital models according to the new position was performed.

**Figure 13.**
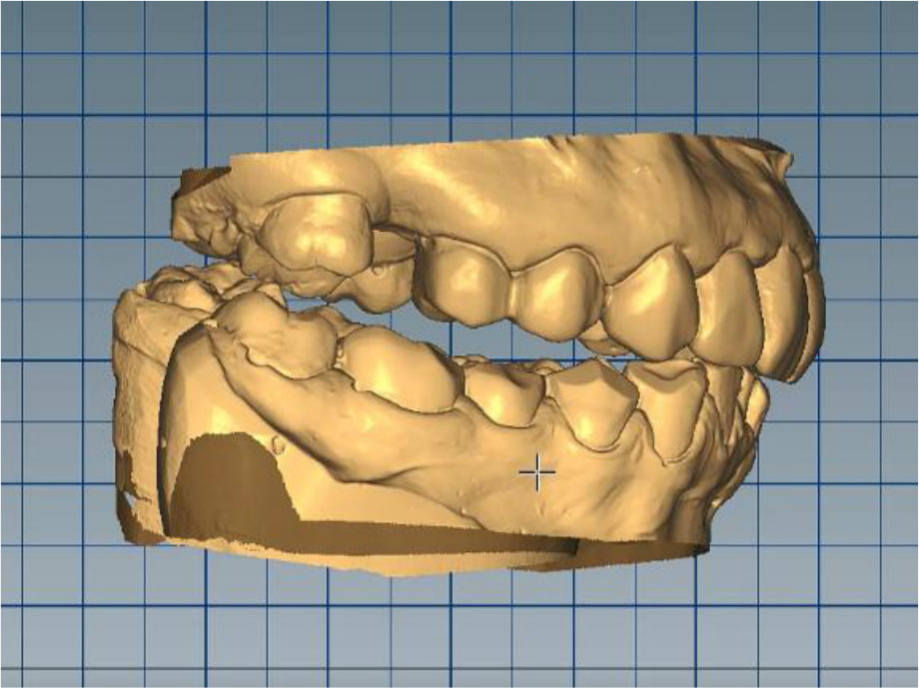
New position of the lower jaw.

**Figure 14.**
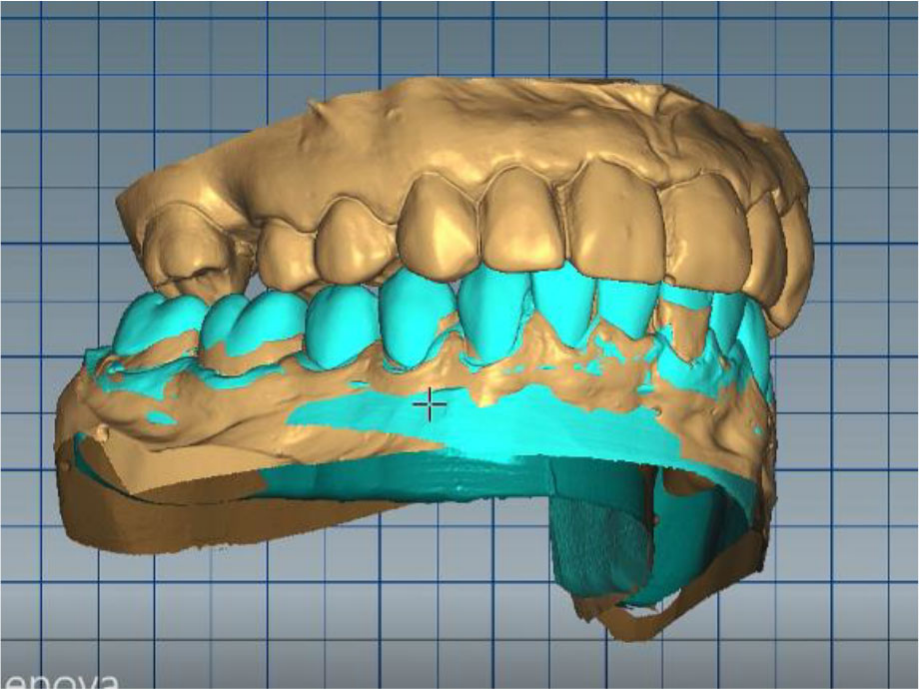
Design of the final restoration.

**Figure 15.**
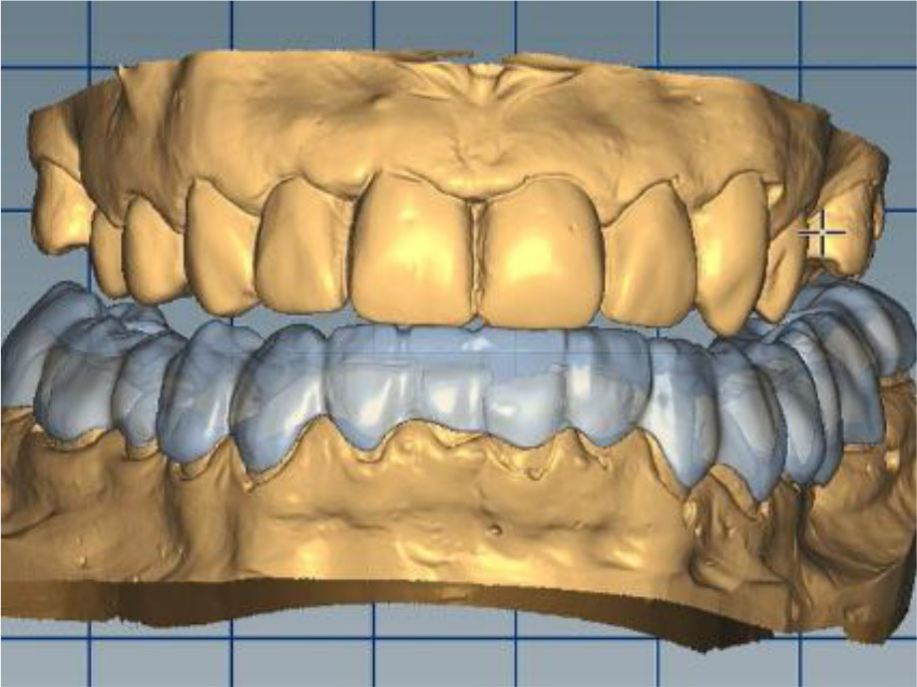
Splint design – anterior protrusion.

**Figure 16.**
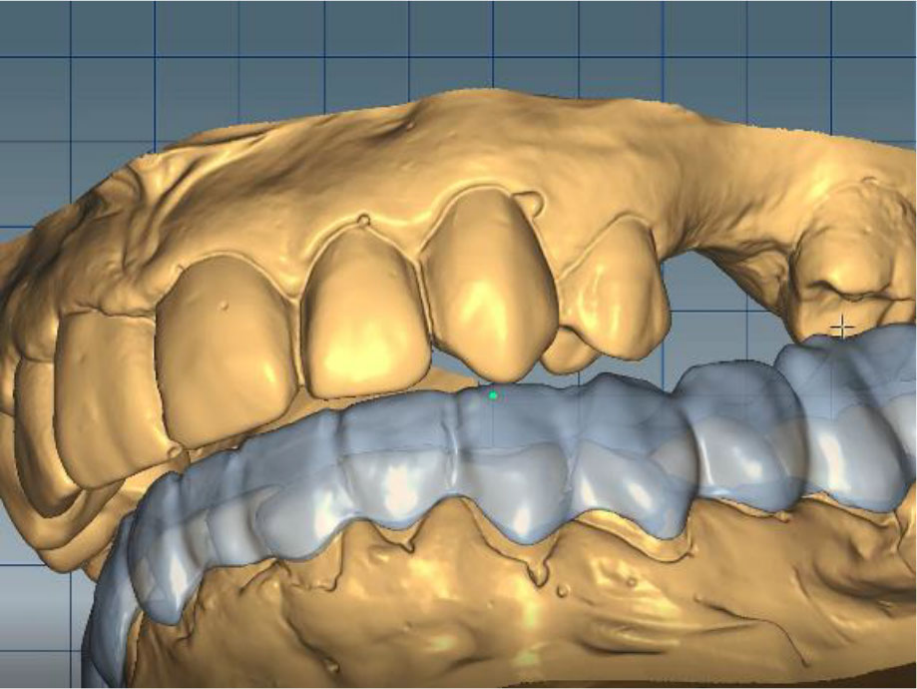
Splint design – lateral protrusion.

In this position, a digital design of the occlusal splint of the lower jaw was made. The occlusal relief of the splint was consistent with the maxillary relief and was made to fix the new position of the jaw so as to relieve TMJ. In forward protrusion, contact was designed only in the area of the incisors, in left and right movements - only canine, retrusive control in premolars and first molars. The splint was designed occlusively with balanced extended surfaces to maintain the occlusion, respectively the joint, in a stable position ( Figures 13- 16).

*Stage 4: Splint placement on the lower jaw and follow-up*

**Figure 17.**
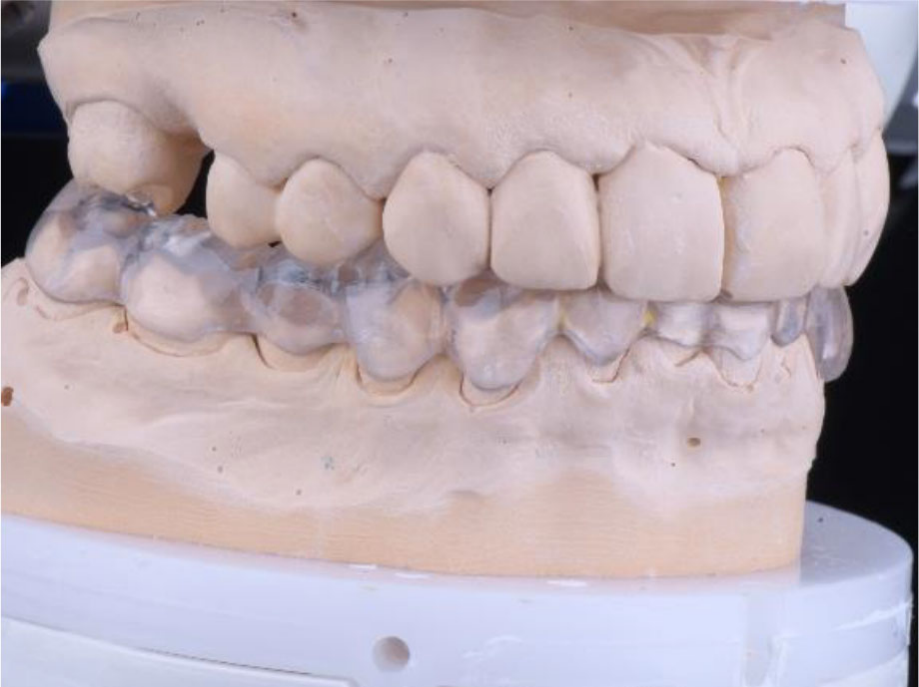
Splint on the model.

**Figure 18.**
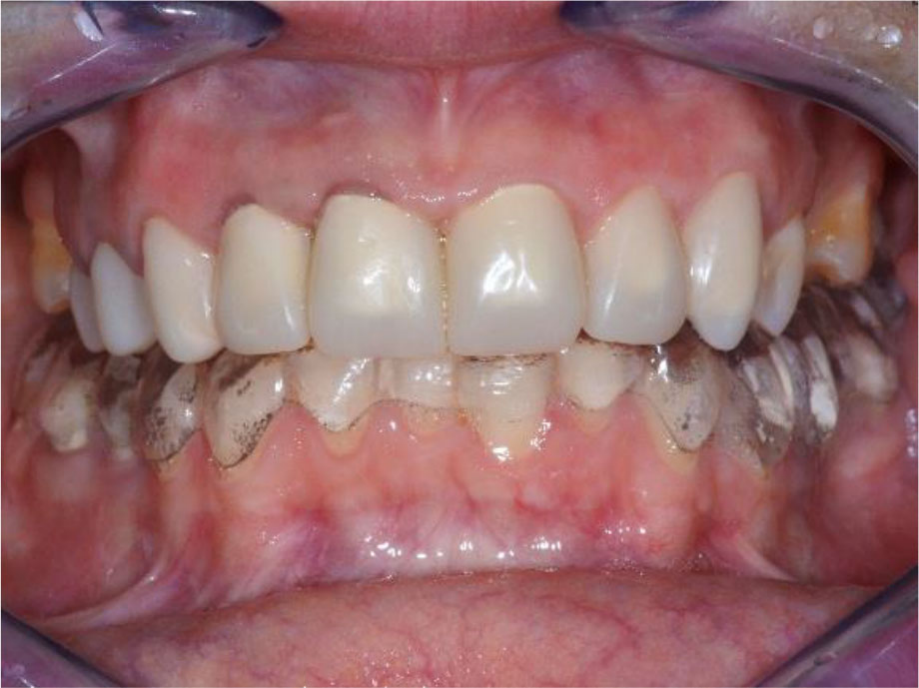
Splint in the patient’s mouth.

The milling splint was checked on an Artex CR articulator (AmannGirrbach). The splint was made from Ceramill Splintec (AmannGirrbach). It was placed in the patient’s mouth and instructions for use and hygiene were given. This splint should be rinsed after each meal, washed with a non-abrasive brush and toothpaste, or use cleaning tablets. The splint made in this way must be worn 24 hours per day for a period of at least 3 months, including during meals and sleep, which guarantees its therapeutic effect ( Figures 17 and 18).

*Stage 5: Morphological planning of the lower jaw and splint replacement*

Preparation for the lower dentition was performed, and two-phase two-stage impressions were taken (ZetaSoft + Oranwash, Zhermack). An intraoral scan was also made. To validate the information, the more accurate of the two models was used (either the direct intraoral scan or the scanned plaster model). The position was fixed by the help of the splint. Temporary constructions of Ceramill TempML were placed and the patient was followed-up for a period of 3 months.

*Stage 6: Completion of the case with ceramic restorations*

**Figure 19.**
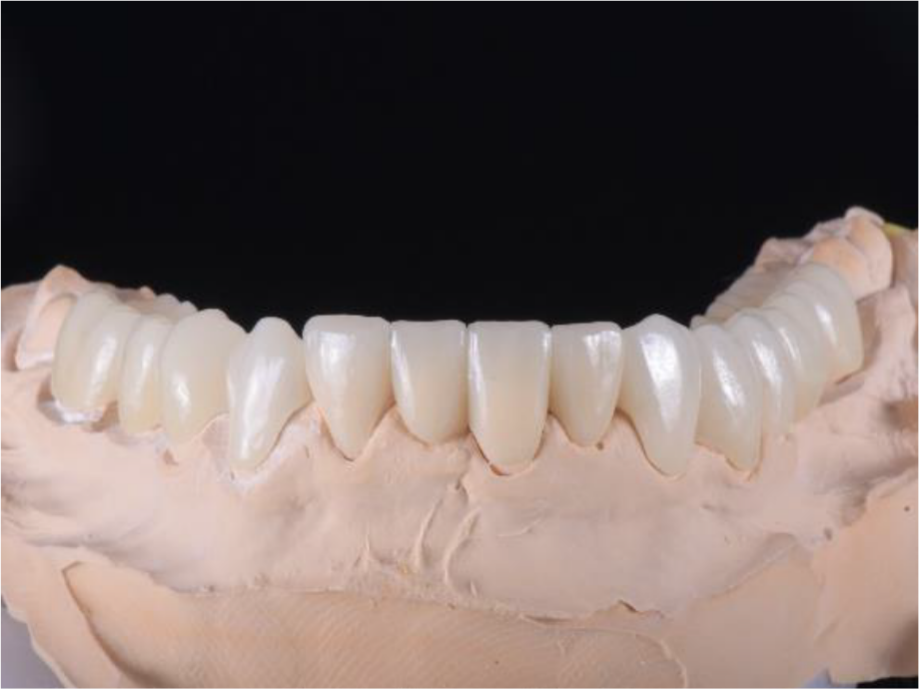
Zirconia prosthesis on the model.

**Figure 20.**
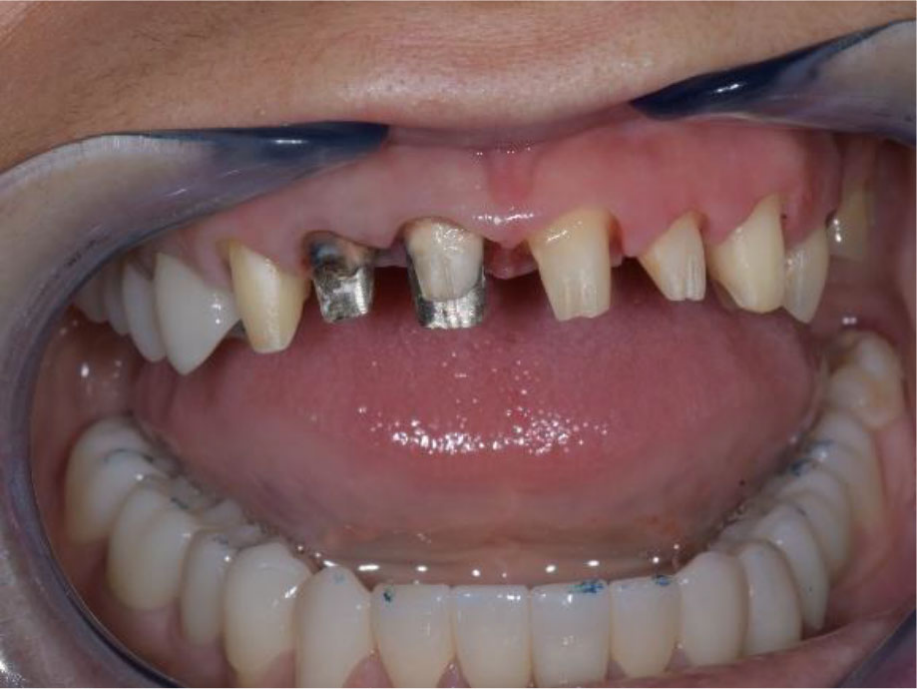
Zirconia prosthesis in the patient’s mouth (try-in procedure).

The patient reported a lack of symptoms of TMJ after 3 months, establishing the therapeutic effect of the splint. We then proceeded to the final prosthesis. All constructions were made of ceramics based on zirconium dioxide, with added yttrium and hafnium oxides. The distal defects of the lower jaw were solved with bridge constructions, and single crowns were placed on the frontal teeth ( Figures 19 and 20).

**Figure 21.**
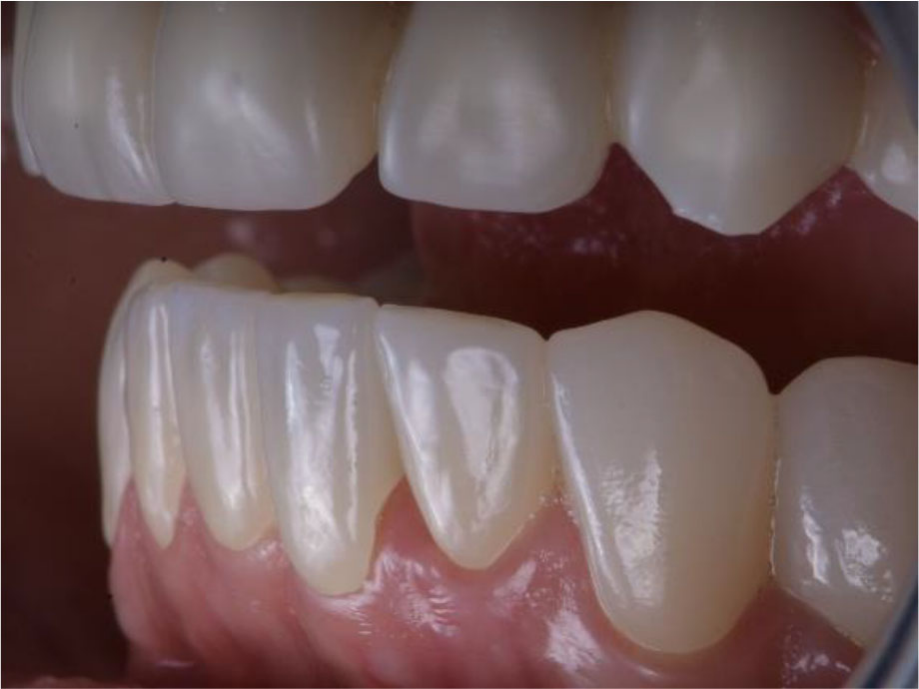
Lower zirconia crowns, upper PMMA during the try-in procedure.

**Figure 22.**
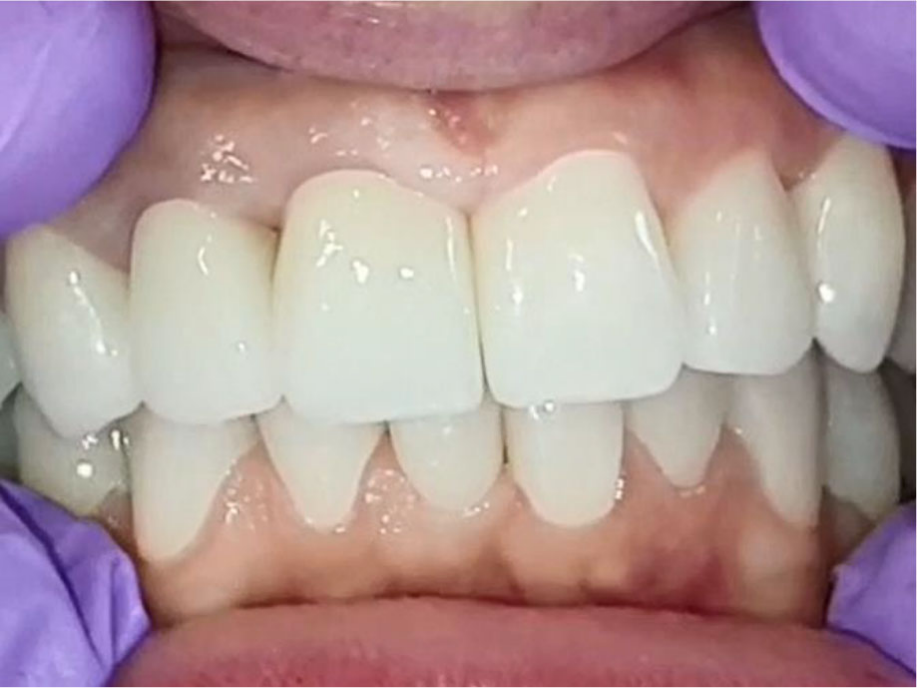
Upper and lower zirconia crowns.

All of the final constructions were made from Ceramill Zolid FX MultiLayer B2 and Glase (AmannGirrbach). For the upper jaw, single zirconia crowns of the canines and block crowns of 11, 12 and 21, 22, respectively, were made, for the distal defects - bridge restorations of zirconia ceramics. The distal structures of both jaws were cemented first to ensure stable occlusion. Then the crowns were fixed almost at the same time in the frontal section ( Figures 21 and 22).

*Follow up and outcomes*

Follow-up was performed at each new step throughout treatment. No adaptation period was observed after the final prosthesis. The patient was scheduled for periodic monitoring at 3 months. No clinical symptoms of the TMJ were found, the patient also did not report.

## Discussion

Splint therapy is the main method of treating bruxism. The case reported here demonstrates a clinical protocol that combines various techniques used previously for bruxism. Digital technologies very accurately recreate clinical situations. They allow changes with an accuracy of 0.01 mm, which is impossible with classical laboratory technologies. The position of the jaws and their fixation in the optimal position can be discussed by sending a photo by email, phone and others. This excludes a "surprise" from the appearance of the final product.

VDO recovery is a major factor that affects muscle hyperactivity. ^1, 2^ Medialization achieves the correct position of the joint head in the joint fossa and reduces the disappearance of joint symptoms. ^11^ Balanced stable occlusion is mandatory. Multiple evenly distributed contacts, as well as extension of the contact surface of the splint with the upper dentition help to obtain this. ^1, 3^ Digital design and the inclusion of models in the digital articulator are particularly helpful and reduce the clinical time for splint adjustment in the patient’s mouth. ^13^ A digital design and CAD/CAM technology provides a splint that is strong enough to be worn continuously, even during meals. This determines great therapeutic effect, as no return to the initial situation can occur. ^16^ The final prosthesis performed on the dentition of both jaws fixes and stabilizes the restored VDO. In addition to therapeutic outcomes, zirconia constructions have a high aesthetic and psycho-preventive effect. ^17, 18^

## Conclusions

The therapeutic protocol presented here, which combined analog and digital measures, achieved the following in our patient: restored defects in the dental arches and hard dental tissues; recovered the normal height of the lower third of the face (VDO); fixed a stable and balanced position of the lower jaw; and repaired the normal physiological position of TMJ.

## Data availability

All data underlying the results are available as part of the article and no additional source data are required.

## Consent

Written informed consent was obtained from the patient to publish this case report and any associated images.
