# Supplementary material for: Case Report: A digital workflow in the treatment of bruxism in a young patient
Source: F1000Res. 2022 Sep 2;10:894. Originally published 2021 Sep 7. [Version 2] doi: 10.12688/f1000research.72961.2 (PMC9445565; doi:10.12688/f1000research.72961.2)
Supplement: Peer review history

# Peer review: Reviewer response for version 2

**DOI:** 10.5256/f1000research.137676.r149344

**Published:** 2022-09-05

**Authors:** Essam Ahmed Al‐Moraissi

**Affiliations:** 1 Department of Oral and Maxillofacial Surgery, Thamar University, Dhamar, Yemen

The authors addressed my comments appropriately

Are enough details provided of any physical examination and diagnostic tests, treatment given and outcomes?

Yes

Is the case presented with sufficient detail to be useful for other practitioners?

Yes

Is sufficient discussion included of the importance of the findings and their relevance to future understanding of disease processes, diagnosis or treatment?

Yes

Is the background of the case’s history and progression described in sufficient detail?

Yes

Reviewer Expertise:

Oral and Maxillofacial Surgery

I confirm that I have read this submission and believe that I have an appropriate level of expertise to confirm that it is of an acceptable scientific standard.

# Peer review: Reviewer response for version 1

**DOI:** 10.5256/f1000research.76575.r146608

**Published:** 2022-08-16

**Authors:** Essam Ahmed Al‐Moraissi

**Affiliations:** 1 Department of Oral and Maxillofacial Surgery, Thamar University, Dhamar, Yemen

Well-written case report showing step by step how authors managed 35 years old female with bruxism. the whole steps and photos were clear and understood. I approved this case report for indexing. Only very minor suggestions to mention the names of abbreviations at the first time in both abstract and text such as PMMA.

Are enough details provided of any physical examination and diagnostic tests, treatment given and outcomes?

Yes

Is the case presented with sufficient detail to be useful for other practitioners?

Yes

Is sufficient discussion included of the importance of the findings and their relevance to future understanding of disease processes, diagnosis or treatment?

Yes

Is the background of the case’s history and progression described in sufficient detail?

Yes

Reviewer Expertise:

Oral and Maxillofacial Surgery

I confirm that I have read this submission and believe that I have an appropriate level of expertise to confirm that it is of an acceptable scientific standard, however I have significant reservations, as outlined above.

# Response

**Published:** 2022-08-18

**Authors:** dobromira shopova

Thank you very much for your review! 

 I will correct the article according to your suggestions!

# Peer review: Reviewer response for version 1

**DOI:** 10.5256/f1000research.76575.r126807

**Published:** 2022-04-21

**Authors:** Hristina Arnautska

**Affiliations:** 1 Faculty of Dental Medicine, Department of Prosthetic Dentistry, Medical University -Varna, Varna, Bulgaria

I think the article is extremely useful and clinically focused. Provides a clear protocol for work in patients with bruxism and TMJ problems associated with altered occlusion. In my opinion, some points should be added to the discussion, which I have noted below, to add a scientific basis for the clinical protocol.

**Some comments:**

- The diagnostic part does not take into account the rotated position of the lower jaw around the condyle, which together with the deep and distal bite is part of the patient's problem list, related to the joint disorder. Figure 3 is the same as figure 1 and it is not required.
- In phase 3 it is not clear what requires optimization of the position of the lower jaw - perhaps it is the laterodeviation of the mandible (which is missing in the diagnosis) that leads to the need for rotation around the condyle.
- At stage 5 the authors explain that they fixed the position of the lower jaw with a splint. Is this the same splint used to fix the joint in the correct position, or is it a new working splint?

In the discussion, in my opinion, there is no information on:

- whether step by step medialization of the jaw is recommended when more than 3 mm medialization is required in a non-growing patient;
- whether only three months with the splint are enough to ensure the stability of the position and how the muscles would affect the occlusion after the removal of the splint. Good occlusal contacts are not enough to fix the position of the lower jaw in non-growing patients, especially with TMJ problems.
- Do they consider it advisable for the patient to continue wearing an occlusal splint for a period of time to allow the muscles to begin to change their muscle tone and to stabilize the new position of the mandible?

Are enough details provided of any physical examination and diagnostic tests, treatment given and outcomes?

Partly

Is the case presented with sufficient detail to be useful for other practitioners?

Yes

Is sufficient discussion included of the importance of the findings and their relevance to future understanding of disease processes, diagnosis or treatment?

Partly

Is the background of the case’s history and progression described in sufficient detail?

Yes

Reviewer Expertise:

I am an orthodontist. I have interests in the field of surgery, prosthetics and biomechanics

I confirm that I have read this submission and believe that I have an appropriate level of expertise to confirm that it is of an acceptable scientific standard.

# Response

**Published:** 2022-08-18

**Authors:** dobromira shopova

Thank you very much for your review!

 I will correct the article according to your notice!
